# A rare malposition of the thoracic venous catheter introduced via the left internal jugular vein

**DOI:** 10.4103/0972-5229.45083

**Published:** 2008

**Authors:** Supradip Ghosh, Himanshu Dewan, Sandip Bhattacharyya

**Affiliations:** **From:** Fortis-Escorts Hospital, Neelam Bata Road, Faridabad, Haryana-121 001, India

**Keywords:** Catheter malposition, left internal jugular vein catheterization, superior intercostal vein cannulation

## Abstract

A rare malposition of central venous catheter in the left superior intercostal vein is described. The diagnostic features and the possible ways to prevent this complication are discussed.

## Introduction

Central venous catheterization is an essential component of modern day critical care. But the insertion of central venous catheters is not free of complications. Numerous complications described both during placement of the catheter and later in the long-term maintenance, are both hazardous to the patients and expensive to treat. Malposition of the catheter tip is one of such complications, which usually involves placement of the catheter in various large tributaries of superior vena cava. This case report illustrates a rare malposition of a central venous catheter tip in a small tributary of left brachiocephalic vein.

## Case Report

A 28-year-old male, was admitted to our ICU with surgical site infection and severe sepsis. Ten days prior to the present admission he underwent ileal resection and ileostomy for ileal perforation with underlying ileocaecal tuberculosis. On examination, he was in respiratory distress with tachypnoea, tachycardia and bilateral crepitations on chest auscultation. Arterial blood gas showed severe hypoxia. Possibilities of fluid overload as a result of aggressive fluid resuscitation and ARDS were considered and it was decided to place a central venous catheter for monitoring of central venous pressure. A catheter was placed through the left internal jugular vein with all aseptic precautions using the Seldinger technique. The catheter was gradually advanced up to the 13 cm mark without difficulty. After free return of venous blood was obtained, the catheter was felt to be placed correctly in the superior vena cava. Only unusual thing observed was patient complaining of left sided back pain on flushing the catheter with heparinized saline.

**Figure 1:**
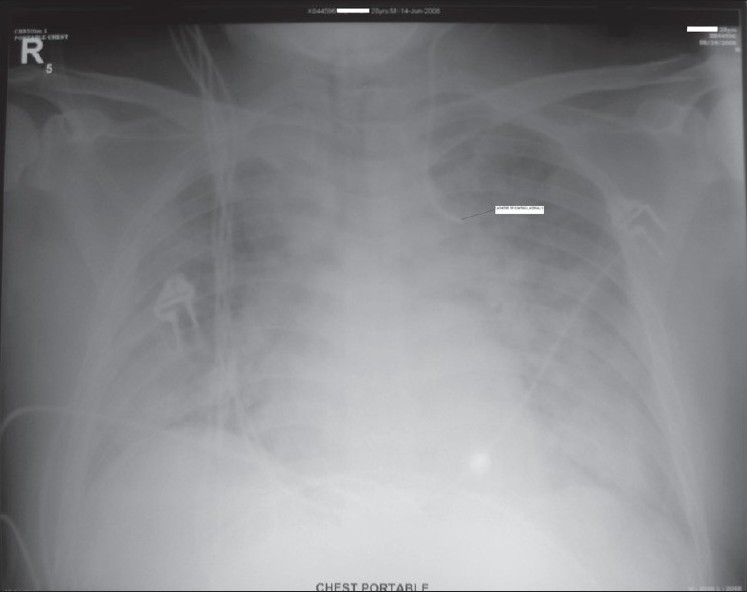
Anteroposterior Chest Radiograph showing central venous catheter lying in the left paramedian location following the aortic knob

An anteroposterior chest radiograph, obtained to confirm the position of the catheter, revealed the left paramedian location of the catheter following the aortic knob and pointing laterally [Figure 1]. The inability to properly position the acutely dypnoeic patient barred us from taking a lateral film. The catheter was removed, as it was considered to be in one of the small tributaries of left brachiocephalic vein and an alternate venous access was obtained subsequently via the right internal jugular vein.

## Discussion

Malposition of central venous catheters was reported to be between 1 to 33 percent by different investigators.[1] These are usually limited within the larger tributaries of the superior vena cava.[1] Malposition of the central venous catheter in the smaller tributaries of the central veins is only rarely reported. Muhm *et al*, with their experience of 2104 central venous catheterization could find only one incidence of misdirected catheter in the smaller tributary.[2]

Thoracic pain syndromes on flushing of misplaced central venous catheters in the smaller tributaries have been described in the literature. Webb *et al,*[1] reported three cases of accidental cannulation of the internal thoracic vein, presenting with retrosternal pain. Patients are reported to complain of midthoracic back pain following cannulation of superior intercostal and azygos venous system.[1–3] Though characteristic chest pain often provides clue to the erroneous catheter position, catheter malposition is more often identified by a post-procedure chest radiograph.[4,5] A properly placed subclavian or internal jugular catheter should run parallel to the shadow of superior vena cava.[6] In the posteroanterior or anteroposterior view, the internal thoracic vein catheter will be located more laterally, a catheter in the superior intercostal vein will follow the aortic knob and pericardiophrenic vein catheter will follow the left cardiac border.[4] In the lateral view catheters in the internal thoracic, pericardiophrenic and superior intercostal veins will occupy the anterior, middle and posterior mediastinum respectively. Placement of the catheter in the remnant of the left-sided superior vena cava should also be kept in mind,[7] in which case the catheter will be in the left paramedian location in the frontal view and will occupy the middle mediastinum in the lateral view.

Because of the lack of experience, the importance of back pain on flushing of the misplaced catheter in our patient was appreciated only retrospectively. The malposition was evident only on review of the postprocedural chest X-ray. The characteristic location of the catheter in the frontal view and the associated classical back pain on flushing the catheter makes the left superior intercostal vein as the most likely position of the catheter. A lateral film and a venogram could have established the exact location of the catheter beyond any doubt.

Because of the longer course and more transverse lie of the left brachiocephalic and more frequent smaller tributaries, malposition of the venous catheter is commoner when cannulation attempt is made via the left brachiocephalic vein rather than its right sided counterpart.[1,4] The malposition of the venous catheter in the smaller tributaries can be prevented by avoiding the left internal jugular/subclavian vein cannulation, limiting the depth of insertion of the guidewire during cannulation and the use of J-tipped guidewire.[8]

After placement of all central venous catheters, a chest radiograph should be obtained. A posteroanterior or anteroposterior film is usually adequate; if not, a lateral view may be taken. If uncertainty still exists, a venogram through the catheter should be performed for precise localization.[9]
